# Antigen Exposure History Defines CD8 T Cell Dynamics and Protection during Localized Pulmonary Infections

**DOI:** 10.3389/fimmu.2017.00040

**Published:** 2017-01-27

**Authors:** Natalija Van Braeckel-Budimir, Matthew D. Martin, Stacey M. Hartwig, Kevin L. Legge, Vladimir P. Badovinac, John T. Harty

**Affiliations:** ^1^Department of Microbiology, Carver College of Medicine, University of Iowa, Iowa City, IA, USA; ^2^Department of Pathology, Carver College of Medicine, University of Iowa, Iowa City, IA, USA; ^3^Interdisciplinary Program in Immunology, Carver College of Medicine, University of Iowa, Iowa City, IA, USA

**Keywords:** CD8 T cells, memory, antigen exposure history, localized infection, influenza

## Abstract

Unlike systemic infections, little is known about the role of repeated localized infections on (re)shaping pathogen-specific memory CD8 T cell responses. Here, we used primary (1°) and secondary (2°) intranasal influenza virus infections of mice as a model to study intrinsic memory CD8 T cell properties. We show that secondary antigen exposure, relative to a single infection, generates memory CD8 T cell responses of superior magnitude in multiple tissue compartments including blood, spleen, draining lymph nodes, and lung. Unexpectedly, regardless of the significantly higher number of 2° memory CD8 T cells, similar degree of protection against pulmonary challenge was observed in both groups of mice containing 1° or 2° memory CD8 T cells. Mechanistically, using pertussis toxin-induced migration block, we showed that superior antigen-driven proliferation and ability to relocate to the site of infection allowed 1° memory CD8 T cells to accumulate in the infected lung during the first few days after challenge, compensating for the initially lower cell numbers. Taken together, the history of antigen exposures to localized pulmonary infections, through altering basic cell biology, dictates dynamic properties of protective memory CD8 T cell responses. This knowledge has important implications for a design of novel and an improvement of existing vaccines and immunization strategies.

## Introduction

CD8 T cells play a crucial role in protection against numerous systemic and localized infections (1–6). Therefore, understanding the formation and maintenance of memory CD8 T cell pool, together with the shaping of its basic biological properties, is crucial for the development of better immunization strategies to combat infectious diseases.

The importance of understanding how antigen (pathogen) stimulation history impacts properties of memory CD8 T cell pool has been emphasized recently (7–9). Importantly, it has been shown that multiple antigen exposures to systemic infection dramatically change the gene expression profile of memory CD8 T cells, thus reshaping their phenotypic and functional properties (8). Furthermore, it has been shown, using a LCMV systemic infection model, that antigen exposure history defines the protective capacity of memory CD8 T cells, by determining specific cell intrinsic factors (10).

Unlike systemic infections, the basic understanding of biology of memory CD8 T cells induced upon localized infections is limited to a single-exposure model or early time after challenge (secondary) infection (4, 11–14). For example, a study focusing on comparison between primary effector and memory CD8 T cells induced after influenza infection revealed similar relative cytotoxic potential between these different cell types (15). On the other hand, whether and how functional properties of memory CD8 T cells induced by localized (influenza) infections are altered by multiple exposures to cognate antigen is not known. As numerous localized infections, such as pulmonary virus infections (influenza, RSV, adenovirus, etc.), display either a seasonal or reoccurring nature (16, 17), study of CD8 T cell responses induced by such infections in the context of multiple antigen encounters is highly important. Addressing these major knowledge gaps could provide valuable information required for rational design of vaccines and/or immunization procedures.

Here, we used primary (1°) and secondary (2°) influenza A virus (IAV) infection of mice as a model to study the fundamental characteristics of memory CD8 T cells generated during localized pulmonary infection. The study showed that, similarly to systemic infections, history of exposure to localized infections imprints changes in the numerical, phenotypic, and functional properties of the memory CD8 T cell pool. Importantly, some of these intrinsic properties, such as proliferation capacity and the ability to relocate to the site of infection, dictate the cellular dynamics and localization, thus determining the protection against subsequent pulmonary challenge.

## Materials and Methods

### Ethics Statement

Female C57Bl/6 mice, 6–8 weeks old, were purchased from the National Cancer Institute (Fredericksburg, MD, USA). The study protocol was approved by University of Iowa Animal Care and Use committee. The animals were treated and handled in accordance with guidelines established by the committee.

### Influenza Strains and Generation of Memory CD8 T Cells

All IAV infections were performed while mice were under the influence of ketamine/xylazine anesthesia. Virus inoculum was intranasally (IN) delivered in PBS in a final volume of 50 µl.

Secondary memory (2°M) CD8 T cell responses were generated in C57/Bl6 mice in two steps. First, mice were IN infected with 7 × 10^3^ TCID_50_ (median tissue culture infective dose) of A/H3N2/X31. Seventy days later (D0), mice were exposed to secondary IN infection with 10^4^ TCID_50_ of A/H1N1/S12a (SEQ12) (PR8 escape mutant). The S12a mutant virus contains 12 amino acid substitutions in the globular domain of hemagglutinin (HA). It was derived from PR8 virus through 12 sequential selection steps in the presence of anti-HA monoclonal antibodies. This approach allowed for generation of HA drifted virus strain, with no effect on other antigenic determinants (18). Alignment of sequences derived from S13 mutant virus (a descendant of S12a) and wt PR8 showed absence of variation in sequences encoding nucleoprotein (NP) and polymerase acidic (PA) proteins bearing target CD8 T cell epitopes (18).

Primary memory (1°M) CD8 T cell responses were generated on D0 by IN infection with X31 virus. 1°M and 2°M CD8 T cells were analyzed 70–90 days postinfection. For the purpose of longitudinal analysis of the NP_366_-specific response, blood was obtained on days 10, 50, and 100 postinfection and analysis performed as previously described (8, 9).

### Pulmonary Challenge and Lung Virus Titers

Protection against pulmonary virus infection was assessed 70–90 days post-last IAV exposure. For this purpose, 1°M and 2°M mice were exposed to IN challenge with 10^7^ or 10^8^ PFU of recombinant vaccinia virus (Vac) expressing full-length influenza NP (Vac-NP) or mock infected with Vac-OVA (both viruses are obtained from Dr. Jack Bennink, Laboratory of Viral Diseases, National Institute of Allergy and Infectious Diseases). Lungs were harvested 3 days post-challenge, homogenized, frozen, and kept at −80°C until used for titration. For determining Vac virus titers, BSC-40 cells were infected with 10× dilutions of lung supernatant in Iscove’s DMEM medium supplemented with 50 µg/ml gentamicin, 100 U/ml penicillin, and 100 µg/ml streptomycin. Cells were incubated with the lung virus samples for 1 h at 37°C, with occasional rocking and afterward overlaid with complete DMEM. After in total 48 h incubation at 37°C, medium was aspirated, cells were fixed with 7% formaldehyde and stained with 0.5% crystal violet in 10% methanol, and plaques were counted (10, 19, 20).

### Intravascular Exclusion and Preparation of Lung Samples

To distinguish cells in the lung vasculature from those imbedded in the lung parenchyma, 3 min prior to sacrifice mice were intravenous (IVS) injected with 2 µg of anti-CD45.2 antibody (clone 104, BioLegend, San Diego, CA, USA) in PBS (21). Harvested lung was cut into small pieces and incubated for 1 h at 37°C in digestion medium composed of 125 U/ml collagenase and 0.1 mg/ml DNAse. After incubation, tissue was processed into single-cell suspension using cell strainers and leukocytes enriched by centrifugation in 35% Percoll in HBSS. Red blood cells were lysed using Vitalyze.

### Tetramer Staining and Antibodies

Antigen-specific CD8 T cells were identified by labeling with D^b^/NP_366_ or D^b^/PA_224_ *in house* prepared tetramers for 1 h at 4°C (22). Tetramer staining was followed by surface staining with appropriate antibody cocktails for 20 min at 4°C. Surface markers were stained using following antibodies: anti-CD8 (clone 53-6.7, BioLegend), anti-CD90.2 (clone 30-H12, BioLegend), anti-CD45.2 (clone 104, BioLegend), anti-CD103 (clone 2E7, BioLegend), anti-CD69 (clone H.12F3, BioLegend), anti-KLRG-1 (clone 2F1, eBioscience, San Diego, CA, USA), anti-CD127 (clone A7R34, BioLegend), anti-CX3CR1 (clone SA011F11, BioLegend), anti-CXCR3 (clone CXCR3-173, BioLegend), and anti-CD49a (clone Ha31/8, BD Pharmingen). Intracellular cytokine staining was performed using anti-IFNγ (clone XMG1.2, BioLegend), anti-TNF (clone MP6-XT22, BioLegend), and anti-IL2 (clone JES6-5H4, BioLegend) antibodies. Proliferation of CD8 T cells was assessed by intracellular staining with anti-Ki67 (clone MOPC-21, BD Pharmingen). Flow cytometry data were acquired using LSRFortessa (Becton Dickinson, Rutherford, NY, USA) and analyzed using the FlowJo software (Tree Star Inc., Ashland, OR, USA).

## Results

### Experimental Model

The major aim of this study is to investigate the influence of repeated localized pulmonary infections on shaping the pathogen-specific memory CD8 T cell compartment. For this purpose, we took advantage of a well-established mouse model of IAV infections (23–25) and generated virus-specific 1°M and 2°M CD8 T cells by exposing naive *wt* C57Bl/6 mice to one or two intranasal IAV infections, respectively. The selected virus strains (H3N2 X31 and H1N1 S12a) share some common gene segments that encode virus core proteins (e.g., NP and PA protein) and thus CD8 T cells epitopes (NP_366_ and PA_224_), enabling successful boosting or primary memory CD8 T cell response by secondary infection (26, 27). This approach allowed us to study and compare the development of endogenous 1°M and 2°M CD8 T responses in an intact, *wt* host.

**Figure 1:**
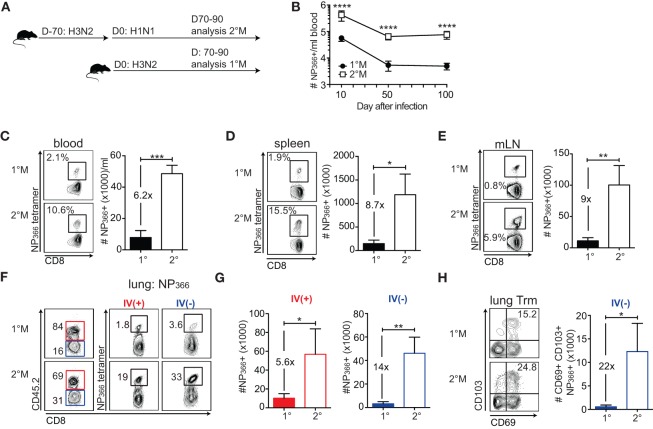
**Secondary infection induces memory CD8 T cell responses of a superior magnitude compared to a primary infection**. **(A)** Naive C57Bl/6 mice were exposed to a single IN infection with X31 H3N2 influenza A virus (IAV) (1°M). Alternatively, mice were infected with X31 H3N2 and 70 days later exposed to a secondary infection with S12a H1N1 IAV (2°M). From 70 to 90 days after the last IAV infection, groups of mice were sacrificed, organs were harvested, and analysis of memory CD8 T cell responses was performed. **(B)** Kinetic of NP_366_-specific CD8 T cell response followed using tetramer staining in blood of 1°M and 2°M CD8 T cell-bearing mice (*n* = 15 mice/time point). Representative of two independent experiments. Error bars represent mean ± SEM. Unpaired *t* test; ****p < 0.0001. **(C–E)** Snap shot analysis of NP_366_-specific CD8 T cell response measured in various tissues 70–90 days after the last IAV infection; blood **(C)**, spleen **(D)**, and mediastinal lymph nodes (mLN) **(E)** of 1°M (closed bars) and 2°M CD8 T cell-bearing mice (open bars). Left—representative plots, right—summary bar graphs. **(F)** Representative plots and **(G)** summary bar graphs of the magnitude of NP_366_-specific CD8 T cell response measured in the lung vasculature (red, IV+) or lung parenchyma (blue, IV−) of 1°M (open bars) and 2°M CD8 T cell-bearing mice (open bars). **(H)** Numbers of NP_366_-specific lung T_rm_ cells measured in 1°M (closed bars) and 2°M CD8 T cell-bearing mice (open bars). Left—representative plots, right—summary bar graphs (*n* = 4 mice/group). Representative of three independent experiments. Error bars represent mean ± SEM. Unpaired *t* test; *p < 0.05, **p < 0.01, ***p < 0.001.

To be able to collect samples and perform analysis of both 1°M and 2°M CD8 T cells at the same time and this way minimize the variability between assays, we adopted the infection scheme depicted in the Figure 1A. Namely, 2°M CD8 T cell responses were generated in two steps: primary infection with H3N2 X31 followed 70 days later by secondary infection with H1N1 S12a. At the same time of secondary infection, 1°M CD8 T cell responses were generated in a separate group of mice by exposure to H3N2 X31. Mice harboring 1°M or 2°M CD8 T cell responses were sacrificed in groups of 4–5 mice on days 70–90 after the last infection, and analyses were performed. Longitudinal analysis of NP_366_-specific response was performed in a separate group of mice, and blood for this purpose was collected at days 10, 50, and 100.

### Secondary Localized Infection Induces CD8 T Cell Responses of Superior Magnitude

To study how repeated exposures to localized pulmonary infection influence the magnitude of memory CD8 T cell response, we focused our analysis on 1°M and 2°M CD8 T cell responses specific for two major epitopes: NP_366_ (Figures 1B–H) and PA_224_ (Figure S1 in Supplementary Material). Antigen-specific CD8 T cells were enumerated using MHC-I tetramer staining. Longitudinal analysis of the NP_366_-specific response in blood shows that secondary localized infection generates CD8 T cell responses that are of superior magnitude (~10× higher) compared to 1°M responses. Importantly, these numerical differences are conserved throughout the memory phase (D50 and D100) (Figure 1B).

Snapshot analysis of NP_366_-specific CD8 T cell responses performed 70–90 days after the last infection in multiple tissues confirmed our observation from the blood compartment (Figure 1C). In both spleen and draining mediastinal lymph nodes, 2°M NP_366_-specific responses exceeded 1°M responses by ~8× fold (Figures 1D,E). Similar results were observed with PA_224_-specific 1°M and 2°M CD8 T cell responses (Figures S1A–C in Supplementary Material).

Although antigen-specific CD8 T cell numbers measured in blood and lymphoid compartment can be used as a general assessment of the magnitude of virus-specific CD8 T cell response, characterization of the response in the lung, the target organ of respiratory pathogens, is essential. Furthermore, a major focus has recently been put on the population of tissue resident memory CD8 T cells (T_rm_) as the important mediators of protection against localized infections (6, 28, 29). As lung perfusion has shown to be inefficient in removing all the cells trapped in the lung vasculature and has the capacity to destroy lung parenchyma, we administered an intravascular (IV) CD45.2 stain to discriminate between CD8 T cells imbedded in the tissue parenchyma (IV−) from those in the capillaries (IV+) (Figure 1F) (30, 31). Results of NP_366_ tetramer staining of lung cells clearly show a large difference in magnitude of 1°M and 2°M CD8 T cell responses in both the IV+ and IV− lung compartment. Relative abundance of NP_366_-specific CD8 T cells, presented in Figure 1F as % of total IV+ or IV− CD8 T cells, was uniformly higher in lungs of mice exposed to two sequential infections compared to the lungs of mice exposed to a single infection. The difference in absolute numbers of NP_366_-specific 1°M and 2°M CD8 T cells (Figure 1G) was 14× and 5.6× in the lung parenchyma and the vasculature, respectively. Finally, enumeration of the NP_366_-specific IV− CD8 T cells expressing a conventional T_rm_ phenotype (CD69+CD103+) (32, 33) shows that secondary exposure causes >10× increase in size of T_rm_ population, relative to a single exposure (Figure 1H). A similar pattern was observed with PA_224_-specific CD8 T cells (Figures S1D–F in Supplementary Material).

In summary, the data show that the size of antigen-specific 2°M CD8 T cell compartment dramatically exceeds the size of 1°M compartment at all time points after infection. Although somewhat enhanced in the lung parenchyma, this trend is well preserved, in all the analyzed tissues. Given the notion that the CD8 T cell-dependent protection against a pathogen infection is mainly determined by the magnitude of the response (34, 35), our results suggest that higher-magnitude responses induced by secondary infection could provide superior protection against subsequent exposure to a pathogen expressing cognate antigen.

### 1°M and 2°M CD8 T Cell Responses Show a Similar Level of Control of Pulmonary Virus Infection

**Figure 2:**
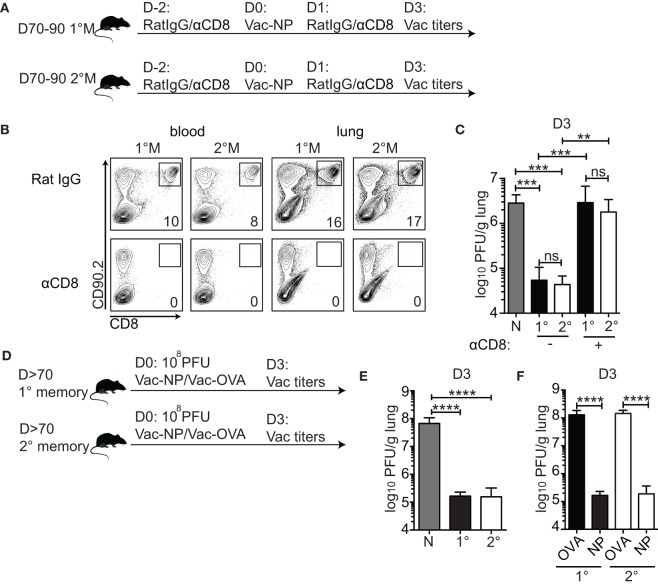
**1°M and 2°M CD8 T cell-bearing mice show similar level of protection against pulmonary challenge with Vac-NP**. **(A)** From 70 to 90 days after the last influenza A virus (IAV) infection, protection against IN challenge with Vac-NP was evaluated in 1°M and 2°M CD8 T cell-bearing mice. Two days prior to and 1 day post-challenge, mice were inoculated intraperitoneally and IN with αCD8 or rat IgG antibodies. Three days post-challenge lung Vac virus titers were measured. **(B)** Administered dose of αCD8 antibody successfully depleted total CD8 T cell population, as shown by the absence of CD8+ cells in peripheral blood and lung in both 1°M and 2°M CD8 T cell-bearing mice. **(C)** Vac-NP titers measured in lungs of naive, 1°M and 2°M CD8 T cell-bearing mice treated with αCD8 or rat IgG antibody. **(D)** From 70 to 90 days post-IAV infection, 1°M and 2°M CD8 T cell-bearing mice were exposed to IN challenge with 10^8^ PFU Vac-NP or Vac-OVA. Mice were sacrificed on day 3 post-challenge, and lung Vac titers were measured. **(E)** Virus titers measured in the lungs of naive, 1°M and 2°M CD8 T cell-bearing mice 3 days after IN challenge with 10^8^ PFU Vac-NP. **(F)** Virus titers measured in lungs of 1°M and 2°M CD8 T cell-bearing mice 3 days after IN challenge with 10^8^ PFU Vac-NP or control Vac-OVA (*n* = 4–5 mice/group). Representative of two to three independent experiments. Error bars represent mean ± SEM. One-way ANOVA multiple comparison; ns, non-significant; **p < 0.01, ***p < 0.001, ****p < 0.0001.

Next, we determined whether the substantial increase in numbers of antigen-specific memory CD8 T cells is associated with superior protection of the lung against localized challenge. In order to determine CD8 T cell-mediated protection and avoid the contribution of non-CD8 T cell protective responses potentially induced by previous infections (36–38), we exposed mice to IN challenge with a high dose (10^7^ PFU) of recombinant vaccinia virus expressing full-length influenza PR8 NP (Vac-NP) (Figure 2A).

To formally confirm the role of CD8 T cells as the only mediators of pulmonary protection in this challenge model, mice were intraperitoneally (IP) and IN inoculated with either CD8-depleting antibody or control rat IgG. As shown in Figure 2B, this regime was sufficient to deplete systemic (peripheral blood) and lung-residing CD8 T cells (Figure 2B). Pulmonary protection was assessed by measuring lung virus titers 3 days after infection—time point experimentally determined as the peak of the viral load (data not shown). Both 1°M and 2°M CD8 T cell-bearing mice were able to control the lung Vac-NP infection (Figure 2C). Although we cannot completely rule out the contribution of non-CD8 T cell-mediated cross-protective immunity against vaccinia infection (39), the complete loss of control of lung viral load in both groups of mice after depletion of CD8 T cells strongly suggests that CD8 T cells are the main mediators of the observed pulmonary protection. To our surprise, despite the large difference in magnitude of memory CD8 T cell responses, both 1°M and 2°M CD8 T cell-bearing mice displayed similar control of lung virus burden (Figure 2C). In both groups, we observed a reduction in virus titers by ~100× relative to naive controls. To rule out the possibility that the challenge dose of Vac-NP was not high enough to reveal potential differences in protection between mice containing 1°M and 2°M CD8 T cells, we increased the challenge dose by 10-fold (10^8^ PFU) (Figure 2D). To make the experimental controls more stringent, we infected groups of 1°M and 2°M CD8 T cell-bearing mice with Vac virus expressing chicken ovalbumin (Vac-OVA) as a non-relevant antigen (Figure 2D). Interestingly, in spite of the 10× increase in challenge dose of Vac-NP virus, similar level of pulmonary protection (>500× reduction relative to naive controls) was observed in both 1°M and 2°M CD8 T cell-bearing mice (Figure 2E). Importantly, both groups of mice challenged with Vac-NP showed a statistically significant decrease in lung virus titers relative to the infection- and age-matched controls challenged with Vac-OVA (Figure 2F).

Thus, despite the superior magnitude of 2°M CD8 T cell response observed in all the tissue compartments, the level of protection against localized pulmonary challenge provided by 1°M and 2°M responses is unexpectedly similar when assessed 70–90 days postinfection. This finding suggests that, in addition to sole numbers of memory CD8 T cells, other cell intrinsic factors play an important role in shaping the protection against localized pulmonary challenge.

### 1°M and 2°M CD8 T Cells Show Similar Phenotypic and Functional Properties

**Figure 3:**
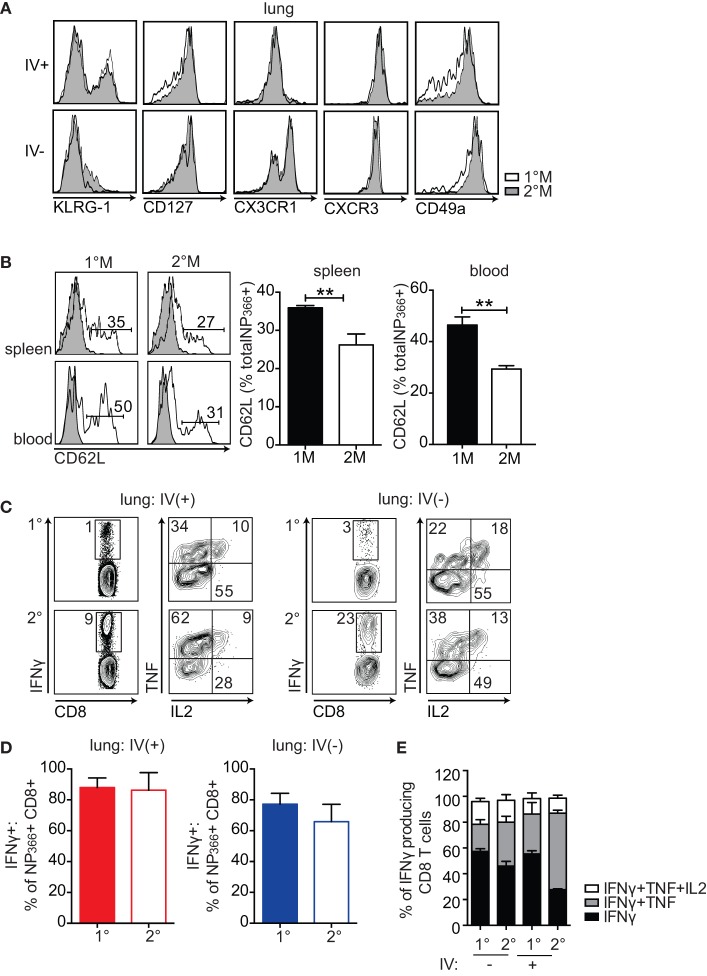
**1°M and 2°M CD8 T cells display similar phenotypic and functional profile**. **(A)** Representative plots (left-to-right) of KLRG-1, CD127, CX3CR1, CXCR3, and CD49a staining of NP_366_-specific CD8 T cells from lung vasculature (IV+) and lung parenchyma (IV−) of 1°M (white) and 2°M CD8 T cell-bearing mice (gray) performed 70–90 days post-last influenza A virus (IAV) infection. **(B)** Representative plots (left) and cumulative bar graphs (right) of CD62L expression evaluated in 1°M or 2°M NP366-specific CD8 T cells isolated from spleen (top panels) and blood (bottom panels) 70–90 days post-last IAV infection. Error bars represent mean ± SEM (n = 4 mice/group). Unpaired t test. **p < 0.01. Representative of 2 independent experiments. **(C)** Cells isolated from lungs of 1°M and 2°M CD8 T cell-bearing mice 70–90 days post-IAV infection were cultured *ex vivo* in presence of EL-4 cells coated with NP_366_ peptide. IV administration of CD45.2 3 min prior to sacrifice allowed for discrimination between lung vasculature and parenchyma. Production of IFNγ, TNF, and IL2 was assessed by intracellular staining. Representative plots of IFNγ (left) and TNF/IL2 staining (gated on IFNγ+; right) of *ex vivo* peptide-restimulated cells derived from lung vasculature (IV+) or lung parenchyma (IV−). **(D)** NP_366_-specific CD8 T cells were enumerated by tetramer staining performed on a separate sample from the same lung cell suspension, as activation of CD8 T cells induces downregulation of the TCR and does not allow for accurate enumeration. Percentage of 1°M and 2°M NP_366_-specific CD8 T cells derived from lung vasculature (IV+) or lung parenchyma (IV−) producing IFNγ as a response to *ex vivo* peptide restimulation (*n* = 4 mice/group). Representative of two independent experiments. Error bars represent mean ± SEM. Unpaired *t* test. No significant differences. **(E)** Cumulative data of single (black, IFNγ), double (gray, IFNγ + TNF), and triple (white, IFNγ + TNF + IL2) cytokine-producing CD8 T cells relative to the total IFNγ-producing CD8 T cells derived from lung vasculature (IV+) or lung parenchyma (IV−) of 1°M and 2°M CD8 T cell-bearing mice (*n* = 3 mice/group). Representative of three independent experiments. Error bars represent mean ± SEM. Multiple comparison two-way ANOVA. *p < 0.05, **p < 0.01, ***p < 0.001, ****p < 0.0001. Statistics: IFNγ 1°M (IV−)–2°M (IV+): *****, 2°M (IV−)–2°M (IV+): **, 1°M (IV+)–2°M (IV+): ***, IFNγ + TNF 1°M (IV−)–2°M (IV−): *, 1°M (IV−)–2°M (IV+): ****, 2°M (IV−)–2°M (IV−): ****, and 1°M (IV+)–2°M (IV+): ****.

To address the possibility that secondary infection induces critical phenotypic and/or functional changes of memory CD8 T cells, this way diminishing their protective capacity, we performed detailed analysis of the memory CD8 T cell pool isolated from lungs of 1°M and 2°M mice 70–90 days post-last infection. Phenotypic characterization evaluating effector (T_em_) vs central memory (T_cm_) properties (KLRG-1, CD127), trafficking (CXCR3), response to inflammatory chemokines (CX3CR1), and tissue localization (CD49a) (8, 40, 41), revealed no major difference between memory CD8 T cells isolated from lungs (IV+ or IV−) of 1°M and 2°M CD8 T cell-bearing mice (Figure 3A). Of note, secondary IAV infection, similar to systemic infections, induced slower acquisition of CD62L expression (T_cm_ properties) in memory spleen- and blood-derived CD8 T cells (Figure 3B). At the same time, both 1°M and 2°M lung-residing CD8 T cells remained CD62L^lo^ (data not shown).

Furthermore, the functional characteristics of lung-derived memory CD8 T cells were assessed by measuring production of IFNγ, TNF, and IL2 after *ex vivo* NP_366_ peptide stimulation. As depicted in Figure 3C, we observed no major difference in functionality of 1°M and 2°M cells, as they were equally able to produce IFNγ, TNF, and IL2 upon peptide restimulation. Importantly, normalizing the numbers of IFNγ-producing CD8 T cells to total number of NP_366_-specific CD8 T cells enumerated by tetramer staining revealed that similar percentage of antigen-specific 1°M and 2°M CD8 T cells produce IFNγ after *ex vivo* peptide restimulation (Figure 3D). Finally, a similar fraction of CD8 T cells capable of producing 1, 2, or 3 cytokines upon *ex vivo* peptide restimulation was observed in both 1°M and 2°M CD8 T cell-bearing mice (Figure 3E). Thus, the similar level of localized pulmonary protection observed in mice harboring 1°M and 2°M CD8 T cells was not due to altered phenotype or compromised functionality of 2°M CD8 T cells.

### Primary Memory CD8 T Cells Compensate for the Initial Lower Numbers by Higher Proliferation Rate and Superior Relocation to the Infected Lung

**Figure 4:**
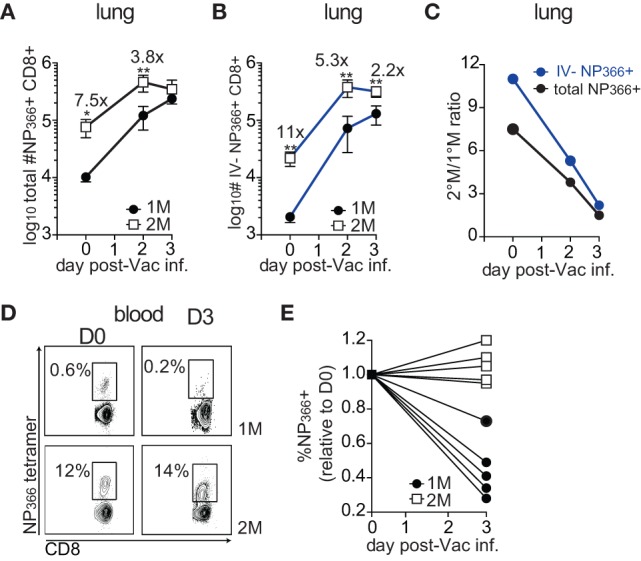
**Early after the onset of the challenge infection 1°M CD8 T cell population in the infected lung undergoes dramatic increase**. **(A)** Change in total numbers of NP_366_-specific CD8 T cells isolated from the lung (black line) or **(B)** from the lung parenchyma (blue line) on days 0–3 post-Vac-NP infection (*n* = 3–5 mice/group). Representative of two independent experiments. Error bars represent mean ± SEM. Unpaired *t* test; *p < 0.05, **p < 0.01. **(C)** Ratio between numbers of NP_366_-specific 2°M and 1°M CD8 T cells isolated from the whole lung (black line) or lung parenchyma (blue line) calculated on days 0–3 post-Vac-NP infection. **(D)** Representative NP_366_-specific tetramer staining of cells isolated from peripheral blood of 1°M and 2°M CD8 T cell-bearing mice on D0 and D3 post-Vac-NP infection. Representative of two independent experiments. **(E)** Size of NP_366_-specific CD8 T cell population measured in peripheral blood of 1°M and 2°M CD8 T cell-bearing mice on D3 post-Vac-NP infection relative to the baseline measured at D0. Representative of two independent experiments.

Similar level of pulmonary protection observed in 1°M and 2°M CD8 T cell-bearing mice could alternatively be explained by a substantial increase in numbers of 1°M CD8 T cells in the lung during the course of the challenge infection. This would potentially allow 1°M CD8 T cells to compensate for the initially low numbers. To directly test this hypothesis, we followed the kinetics of the antigen-specific CD8 T cell response upon pulmonary Vac-NP challenge. To this end, 1°M and 2°M CD8 T cell-bearing mice were exposed to IN challenge with 10^7^ PFU of Vac-NP. Small group of mice was sacrificed before infection (D0) to establish a “baseline” NP_366_-specific CD8 T cell response in the lung and peripheral blood. Infected mice were sacrificed either on day 2 or 3 postinfection, and a snapshot analysis of NP_366_-specific CD8 T cell responses in the lung and blood was performed. On day of infection (D0), numbers of total or IV− NP_366_-specific CD8 T cells in lungs of 2°M CD8 T cell-bearing mice were, respectively, 7.5× and 11× higher compared to the lungs of 1°M CD8 T cell-bearing mice (Figures 4A,B). Furthermore, steep increase in numbers of antigen-specific CD8 T cells was observed after the onset of the infection in both groups of mice. Interestingly, while numbers of antigen-specific 1°M cells continued to rise by day 3 postinfection, numbers of 2°M cells reached a plateau by day 2 postinfection. Due to this non-proportional expansion in the population size of 1°M and 2°M CD8 T cells in the lung, initial numerical differences were completely gone (total NP_366_-specific CD8 T cells) or substantially diminished (IV− NP_366_-specific CD8 T cells) by day 3 postinfection (Figures 4A–C). Analyses of NP_366_-specific CD8 T cells in blood revealed that while the size of the 2°M population seemed to be stable, the size of the 1°M population decreased by day 3 postinfection (Figures 4D,E), suggesting possible relocation of 1°M cells from the circulation into the lung compartment.

**Figure 5:**
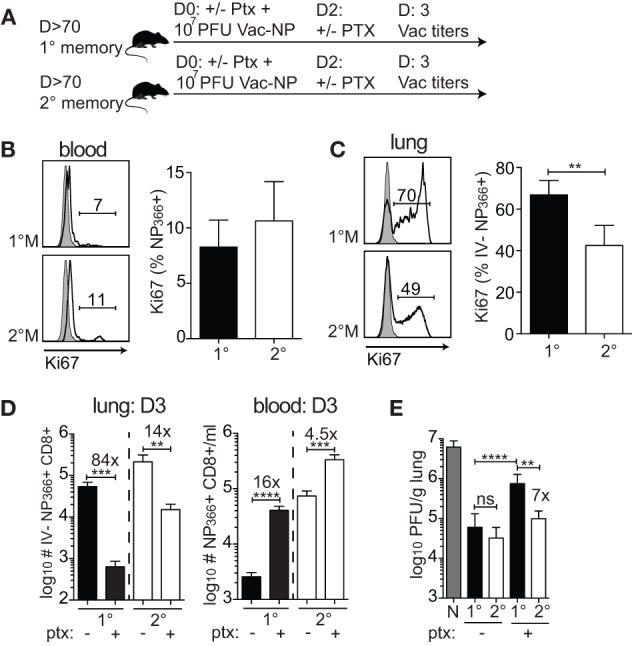
**Superior proliferation and recruitment drive the increase in numbers of 1°M CD8 T cells after the onset of pulmonary challenge**. **(A)** From 70 to 90 days post-influenza A virus infection, 1°M and 2°M CD8 T cell-bearing mice together with naive control mice were exposed to IN challenge with 10^7^ PFU Vac-NP. At the same day, prior to challenge, a group of mice was intraperitoneally injected with pertussis toxin (Ptx). Second dose of Ptx was administered 2 days after the Vac-NP challenge. Mice were sacrificed on D3, and lung virus titers were assessed by plaque assay. **(B)** Representative plots (left) and cumulative data (right) of Ki67 staining of NP_366_-specific CD8 T cells from peripheral blood of 1°M and 2°M CD8 T cell-bearing mice on D3 post-Vac-NP infection. **(C)** Representative plots (left) and cumulative data (right) of Ki67 staining of NP_366_-specific CD8 T cells from lung parenchyma of 1°M and 2°M CD8 T cell-bearing mice on D3 post-Vac-NP infection (*n* = 4 mice/group). Representative of two independent experiments. Error bars represent mean ± SEM. Unpaired *t* test; **p < 0.01. **(D)** Numbers of NP_366_-specific CD8 T cells measured on D3 post-Vac-NP challenge in peripheral blood (left) or lung parenchyma (right) of 1°M and 2°M mice treated or not with Ptx (*n* = 4 mice/group). Representative of two independent experiments. Error bars represent mean ± SEM. Unpaired *t* test; **p < 0.01, ***p < 0.001, ****p < 0.0001. **(E)** Virus titers measured in lungs of 1°M and 2°M CD8 T cell-bearing mice (±Ptx) 3 days after IN challenge with 10^7^ PFU Vac-NP (*n* = 7–8 mice/group). Cumulative results from two experiments. Error bars represent mean ± SEM. Multiple comparison one-way ANOVA. Only relevant statistics are depicted. **p < 0.01, ***p < 0.001, ****p < 0.0001. N-1°M: ****, N-2°M: ****, N-1°MPtx: ***, N-2°MPtx: ****, and 2°M-1°MPtx: ****.

The major increase of 1°M cells in the infected lung could occur as a consequence of the substantial relocation of cells from the blood to the lung and/or due to a superior proliferative capacity of 1°M cells in the lung. To formally test this, 70–90 days after the last IAV infection, 1°M and 2°M CD8 T cell-bearing mice were IN challenged with 10^7^ PFU Vac-NP, and half of them were injected IP with pertussis toxin (Ptx) before (D0) and 2 days after infection to block the access of circulating memory CD8 T cells into the infected lung (Figure 5A) (20). Mice were sacrificed 3 days post-Vac-NP infection, and lung Vac titers and NP_366_-specific CD8 T cell responses were assessed. Proliferation of antigen-specific CD8 T cells was assessed in both blood and lung by staining for Ki67, a cellular marker of proliferation (42). As shown in Figures 5B,C, a substantially higher proportion of 1°M relative to 2°M CD8 T cells had entered one of the active phases of the cell cycle (based on Ki67 expression) in the inflamed lung but not in the blood on day 3 postinfection. Additionally, Ptx treatment caused >80× decrease in numbers of IV− 1°M NP_366_-specific CD8 T cells in the lung, compared to <15× decrease of 2°M. At the same time, Ptx-induced retention of antigen-specific CD8 T cells in blood of 1°M CD8 T cell-bearing mice was more pronounced (16× increase in numbers after Ptx treatment) than in blood of 2°M CD8 T cell-bearing mice (4.5× increase) (Figure 5D).

Importantly, and consistent with the differential impact of Ptx treatment on relocation of 1°M and 2°M CD8 T cells from circulatory to the lung compartment, Ptx-induced block had a greater impact on the control of infection in mice harboring 1°M CD8 T cells relative to those harboring 2°M CD8 T cells. The substantial Ptx-induced decrease in numbers of 1°M CD8 T cells in the lung parenchyma was accompanied by a substantial decrease in protection. As depicted in Figure 5E, ~7× higher virus titers were measured in lungs of 1°M CD8 T cell-bearing mice treated with Ptx as compared to 2°M CD8 T cell-bearing mice. Taken together, the presented data suggest that the superior proliferative capacity of 1°M CD8 T cells in combination with increased recruitment into the infected lung efficiently compensates for the initial lower numbers of antigen-specific 1°M relative to 2°M CD8 T cells, which eventually allows for similar control of localized pulmonary infection in both groups of mice.

Thus, CD8 T cell-mediated protection against localized pathogen infections is not exclusively determined by the magnitude of the response but is also influenced by multiple intrinsic properties (e.g., proliferation and migration to infected tissue) of memory CD8 T cells. History of exposure to localized infections, by shaping such cell properties, determines the protection against subsequent pathogen challenge.

## Discussion

The present study describes how multiple exposures to a localized infection can impact the development of basic biological properties of pathogen-specific CD8 T cells and, as a consequence, protection against subsequent local challenge. Using well-established influenza virus infections of mice as a model of localized pulmonary infections, we compared numerical and functional properties of the infection-induced 1°M and 2°M CD8 T cell pool. Our results show that secondary antigen exposure induces memory CD8 T cell responses of a superior magnitude relative to memory responses generated after a single infection. However, in spite of substantially lower numbers of memory CD8 T cells in all analyzed tissue compartments, mice bearing 1°M CD8 T cells exhibit a similar degree of virus control after pulmonary challenge as 2°M CD8 T cell-bearing mice. Mechanistically, our data show that due to better proliferation and superior capacity to relocate to the site of infection, 1°M CD8 T cells substantially increase their numbers in the infected local tissue, allowing them to successfully control the viral load.

Previous studies using *Listeria monocytogenes* or vesicular stomatitis virus systemic infection models have shown that repeated antigen exposures have a strong impact on the magnitude of memory CD8 T cell responses, by defining the proliferative capacity, responsiveness to environmental cues, and long-term survival (8). Here, we show that, similar to the systemic infection, secondary localized pulmonary infection induces a memory CD8 T cell response of superior magnitude in blood and spleen. Importantly, this trend was also observed in the lung (both vasculature and parenchyma) and draining lymph nodes.

Previous studies comparing the functional capacity of IAV-induced primary effector and memory CD8 T cells showed that although these populations exhibit similar relative cytotoxicity, by the virtue of larger magnitude effector responses displays superior killing of target cells relative to memory cells (15). In contrast, we show that in spite of an almost ~10× difference in numbers of antigen-specific CD8 T cells detected in all the tissues (Figure 1), and no obvious phenotypic and functional impairments of 2°M CD8 T cells (Figure 3), our study revealed a similar degree of pulmonary protection in both 1°M and 2°M CD8 T cell-bearing mice. This suggests that the observed protection does not depend on the sole magnitude of the memory CD8 T cell response but also on CD8 T cell intrinsic factors determined by the antigen exposure history. For example, it has been shown that an equal amount of 1°M and 2°M CD8 T cells exhibit different capacities of protection against acute vs chronic infection. While exhibiting better control of acute infection with LCMV Armstrong, 2°M CD8 T cells failed to control chronic infection with LCMV Clone 13 (10). Furthermore, this study showed that a differential localization of 1°M and 2°M CD8 T cells, shaped by exposure history, determines both virus control in the lymph nodes and the persistent CD8 T cell responses required for successful control of chronic infection. Additionally, it has been shown that repeated antigen stimulations have a dramatic impact on the gene expression profile of memory CD8 T cells (8), and subsequently their basic phenotypic and functional properties, which can substantially impact the capacity to control infections (7, 8). Results of functional and phenotypic characterization of influenza-induced systemic 1°M and 2°M CD8 T cells derived from the spleen corroborate the basic differences described after multiple systemic antigen exposures (7, 8) (data not shown). On the other hand, beside the large numerical differences, 1°M and 2°M CD8 T cells derived from the lung compartment did not exhibit any obvious phenotypic or functional discrepancies. Furthermore, kinetic analysis of the response of 1°M and 2°M CD8 T cells in the lung and blood upon challenge showed that by D3 post-challenge the population of 1°M cells dramatically increases, almost to the level of the 2°M population. The substantially larger proportion of Ki67+ 1°M CD8 T cells detected in the infected lung (Figure 5) suggests that the superior proliferative capacity of 1°M cells contributes, at least partially, to this numerical increase. This finding is in line with the reports showing that the proliferative capacity of CD8 T cells decreases with every subsequent antigen encounter, with naive CD8 T cells exhibiting superior proliferation (7, 8). Administration of Ptx, which blocks the signaling through G protein-coupled receptors, rendering CD8 T cells incapable of responding to inflammatory cues, induced a significant decrease in the accumulation of 1°M CD8 T cells in the infected lung and a subsequent increase in lung virus titers. Additionally, similar to systemic infection, circulating 2°M CD8 T cells induced by localized IAV infections exhibit slower progression toward T_cm_ (CD62L expression) compared to their 1°M counterparts, suggesting the possibility that difference in subset composition of circulating memory CD8 T cells (T_em_ vs T_cm_) might underlie the differences in recruitment to the lung. Together, our data suggest that increased proliferation and superior capacity to relocate to the infected tissue allow 1°M CD8 T cells to substantially increase their numbers in the lung. Thus, number of available antigen-specific memory CD8 T cells is not the only factor determining the outcome of the protection against pulmonary challenge. Here, we present an example of how numerically inferior 1°M CD8 T cells can exploit differential cell properties to establish control of localized viral infection.

In addition to number of encounters with an infectious agent, biology of infection (degree of inflammation, pathogen burden, rate of proliferation, and cell tropism) plays an important role in determining the fundamental properties of memory CD8 T cells (43). Thus, it is likely that repeated pulmonary infections with a pathogen other than influenza virus (e.g., RSV and adenovirus) would leave a different phenotypic and functional imprint on the developing memory CD8 T cells, leading to diminishing or exacerbation of the differences observed between 1°M and 2°M described here. Furthermore, different tissues provide a differential developmental environment, which will additionally impact the development of memory CD8 T cell properties (44). For example, while local infection of the skin leads to generation of stable, long-lived T_rm_ population (45), lung T_rm_ is characterized by rather transient nature (6). Therefore, studies utilizing various localized infection and challenge models will be beneficial to obtain more specific knowledge on the role of antigen exposure history on the development of pathogen-specific memory CD8 T cell responses.

In conclusion, we have shown using an example of a localized pulmonary infection, how antigen exposure history can impact the biology of the memory CD8 T cell responses. Besides altering the magnitude of the response, it leaves an important imprint on intrinsic properties of pathogen-specific CD8 T cells, emphasized in differential cellular dynamics that determine the capacity to control lung infection.

## Author Contributions

NB-B, MM, SH, KL, VB, and JH designed experiments. NB-B performed experiments and data analysis and wrote the manuscript. MM and SH helped obtaining and analyzing some data and reviewed the final manuscript. KL contributed to the discussion and reviewed the final manuscript. VB and JH contributed to writing and editing of the manuscript.

## Conflict of Interest Statement

The authors declare that the research was conducted in the absence of any commercial or financial relationships that could be construed as a potential conflict of interest.

## Supplementary Material

Figure S1 **Influenza PA224-specific CD8 T cell responses measured in various tissues of 1°M and 2°M CD8 T cell-bearing mice**. **(A–F)** Mice were infected as described in Figure 1. PA224-specific CD8 T cell response was detected by tetramer staining. **(A–C)** Snap shot analysis of PA224-specific CD8 T cell response measured 70–90 days after the last influenza A virus infection; blood **(A)**, spleen **(B)**, and mediastinal lymph nodes **(C)** of 1°M (closed bars) and 2°M mice (open bars). Left—representative plots, right—summary bar graphs. **(D)** Representative plots and **(E)** summary bar graphs of the magnitude of PA224-specific CD8 T cell response measured in the lung vasculature (red, IV+) or lung parenchyma (blue, IV−) of 1°M (closed bars) and 2°M CD8 T cell-bearing mice (open bars). **(F)** Numbers of NP_366_-specific lung T_rm_ cells measured in 1°M (closed bars) and 2°M CD8 T cell-bearing mice (open bars). Left—representative plots, right—summary bar graphs (*n* = 4 mice/group). Representative of three independent experiments. Error bars represent mean ± SEM. Unpaired *t* test; **p* < 0.05, ***p* < 0.01, ****p* < 0.001.
