# Supplementary figures and images for: Antigen Exposure History Defines CD8 T Cell Dynamics and Protection during Localized Pulmonary Infections
Source: Front Immunol. 2017 Jan 27;8:40. doi: 10.3389/fimmu.2017.00040 (PMC5269565; doi:10.3389/fimmu.2017.00040)

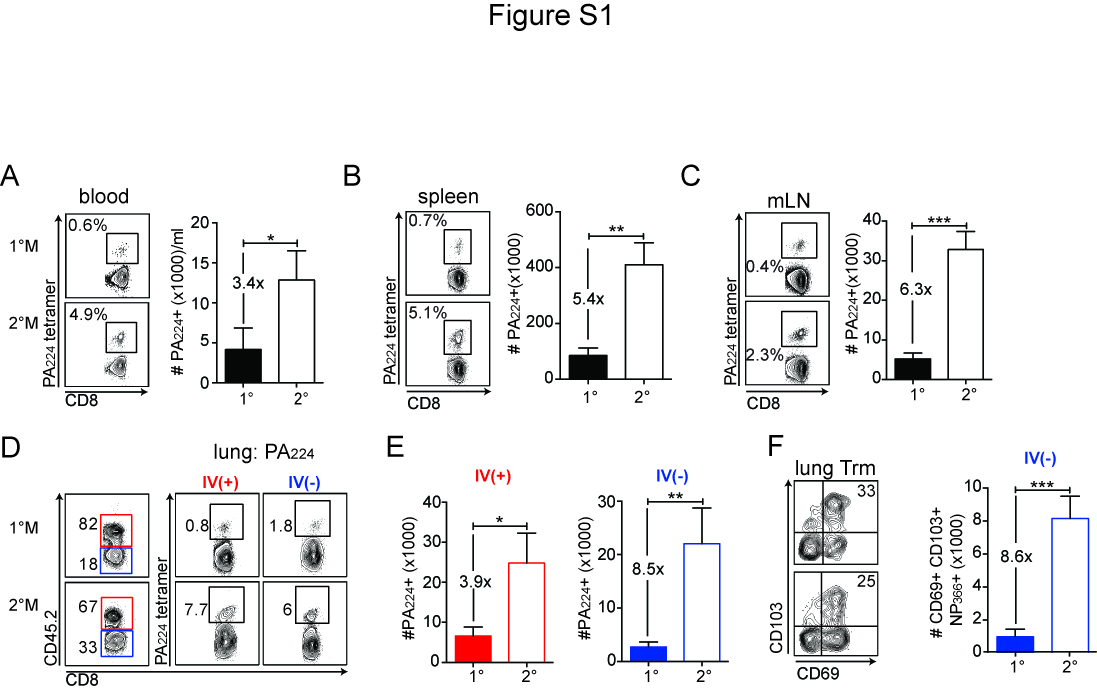

Supplement: Figure S1 — Influenza PA224-specific CD8 T cell responses measured in various tissues of 1°M and 2°M CD8 T cell-bearing mice. (A–F) Mice were infected as described in Figure 1. PA224-specific CD8 T cell response was detected by tetramer staining. (A–C) Snap shot analysis of PA224-specific CD8 T cell response measured 70–90 days after the last influenza A virus infection; blood (A), spleen (B), and mediastinal lymph nodes (C) of 1°M (closed bars) and 2°M mice (open bars). Left—representative plots, right—summary bar graphs. (D) Representative plots and (E) summary bar graphs of the magnitude of PA224-specific CD8 T cell response measured in the lung vasculature (red, IV+) or lung parenchyma (blue, IV−) of 1°M (closed bars) and 2°M CD8 T cell-bearing mice (open bars). (F) Numbers of NP366-specific lung Trm cells measured in 1°M (closed bars) and 2°M CD8 T cell-bearing mice (open bars). Left—representative plots, right—summary bar graphs (n = 4 mice/group). Representative of three independent experiments. Error bars represent mean ± SEM. Unpaired t test; *p < 0.05, **p < 0.01, ***p < 0.001. [file Image_1.TIF]
